# Technical note: Evaluation of three autoanalysers for use in chemical pathology

**DOI:** 10.1155/S1463924690000244

**Published:** 1990

**Authors:** E. M. Narebor

**Affiliations:** 1 Central Laboratory & Blood Bank PO Box 59082 Riyadh 11525 Saudi Arabia; 2 47 St James Road Sutton Surrey SM1 2TG UK

## Abstract

Three autoanalysers, the EPOS 5060, ERIS 6170 and the Analyst, were evaluated for their adequacy for use in laboratories attached to Saudi ‘polyclinics’. All the analysers showed comparable within-batch imprecision. The Analyst was found to be the most useful because it was simple and practical and because its throughput time was faster than the other two analysers. The EPOS 5060 would be more suitable for screening large numbers of samples for single parameters; and the ERIS 6170 essentially suits normal routine chemical laboratory work.


Journal of Automatic Chemistry, Vol. 12, No. 5 (September–October 1990), pp. 189–194

### Technical note: Evaluation of three autoanalysers for use in chemical pathology

**E. M. Narebor†**

*Central Laboratory & Blood Bank, PO Box 59082, Riyadh 11525, Kingdom of Saudi Arabia*

*Three autoanalysers, the EPOS 5060, ERIS 6170 and the Analyst, were evaluated for their adequacy for use in laboratories attached to Saudi 'polyclinics'. All the analysers showed comparable within-batch imprecision. The Analyst was found to be the most useful because it was simple and practical and because its throughput time was faster than the other two analysers. The EPOS 5060 would be more suitable for screening large numbers of samples for single parameters; and the ERIS 6170 essentially suits normal routine chemical laboratory work.*

### Introduction

A number of 'polyclinics' with clinical laboratories were set up in many parts of the Kingdom of Saudi Arabia by the Government in 1984 (these 'polyclinics' are similar in concept to the UK's Health Centres). In the Riyadh health region alone, 80 of the 120 such government-owned 'polyclinics' have clinical laboratories. There are also as many private 'polyclinics' with laboratories in the Riyadh region.

In these 'polyclinics', chemical pathology tests were to be performed with autoanalysers, the results of which were to be reliable and comparable to those produced by the larger hospital laboratories in major cities. An analyser that would suit these 'polyclinics' should be durable; show good, consistent imprecision and accuracy; provide a reasonably quick turn-round time; and should require a very short training period.

The Central Laboratory and Blood Bank in the Central Hospital in Riyadh is a large laboratory. Between 1984 and 1986, various biochemical autoanalysers were evaluated by the Biochemistry Department at the Central Laboratory and Blood Bank. Amongst these were the EPOS 5060, ERIS 6170 (both sold by Eppendorf Geratebau, FR Germany); and the Analyst (Du Pont, USA). Comprehensive studies of the EPOS 5060 and ERIS 6170 have already been published [1, 2]. Only those features of interest to this study are discussed here. So far, no independent study of the Analyst has been published.

The purpose of this study was to determine the adequacy of these autoanalysers for use in 'polyclinics' in Saudi Arabia.

† Current address: 47 St James Road, Sutton, Surrey SM1 2TG, UK.

### Materials and methods

### Instruments

*EPOS 5060 analyser*

This is a bench-top batch analyser. Samples (5–25 μl per test) are loaded via a chain link for assay. Being a batch analyser, reagents are changed manually for each parameter to be assayed, and require between 25–100 μl per test. Reagents of different brands can be utilized without modification (it is an open system). The approximate cost was about 350 000 Saudi Riyals (SR) for the instrument and between 0·05–2·14 SR per test.

*ERIS 6170 analyser*

The ERIS 6170 is a multichannel, discrete analyser of about the same size (1010 × 740 × 1090 mm) and technology as the Hitachi 705 (Boehringer Mannheim GmbH), the autoanalyser in current use in the department for daily routine work. Samples are introduced for assay via a chain link, which is similar to the Epos. However, unlike the Epos, any combination of multiple choice of test assays on any one patient's sample can be analysed. It is also an open system, but during this study reagents from E. Merck (Darmstadt, FR Germany), which were supplied by the agent were used. A built-in computerized quality-control scheme facility is available, however this was not tested nor was it utilized rigorously during this study. The cost price of the instrument was about 600 000 SR and between 0·04–1·40 SR per test.

*The Analyst*

This is a bench-top, compact, sophisticated multichannel discrete profiler analyser. It measures about 216 × 635 × 330 mm and weighs about 19 kg. Reagents are supplied by Du Pont in prepacked disc rotors, which also contain the reaction cuvettes. At the time of this study, the following disc rotors were available.

(*a*) A 12 chem rotor, lot No. 6K1019–12; for the assay of glucose, urea, creatinine, uric acid, cholesterol, calcium, triglycerides, total bilirubin, aspartate transaminase, alanine transaminase, alkaline phosphatase and gamma glutamyl transferase, respectively.

(*b*) A 7 chem rotor, lot No. 565–7; for assaying glucose, urea, creatinine, uric acid, aspartate transaminase, alanine transaminase and cholesterol, respectively.

(*c*) A 3 chem rotor, lot No. 6B1005–3; for glucose, urea, and creatinine assays respectively.

(*d*) A 1 chem rotor, lot No. 6J1006G–1; for glucose assays.

0142–0453/90 $3.00 © 1990 Taylor & Francis Ltd.

All chem rotors used in this study still had at least 9 months' shelf-life before their recommended expiry dates. It is a completely closed system with respect to reagent utility. However, it requires only 90 μl of serum for the assay of all the parameters in the 12 chem rotor. The Analyst does not require daily calibration like most other multichannel discrete autoanalysers. It also has a built-in quality-control system, but this facility was neither tested nor used rigorously during this study. The Analyst costs about 40 000 SR and approximately 1–2·5 SR per test.

*Quality control*

Throughout this study two sets of quality-control (QC) assessment schemes were maintained. One set was physiological and pathological internal (QC) sera supplied by Biotol (rue de Foin, Paris, France), and the other set was supplied by the manufacturers' agents (E. Merck, Darmstadt, FR Germany; Du Pont, Biomedical Product Department, Wilmington, USA). Alternating physiological and pathological QC sera from our internal set and the agents' set were assayed at the beginning and end of each batch. The QC sera were also assayed at random intervals amongst test samples in within-batch analyses. The coefficient of variation of these quality-control sera are shown in table 3.

*Table 1(a). Mean accuracy values compared with Biotrol (commercial internal quality control serum). N = 50.*

| | | Biotrol | EPOS | ERIS | Analyst |
|---|---|---|---|---|---|
| Glucose | 1 | 5·3 | 5·2 | 5·0 | 5·3 |
| (mmol/l) | 2 | 11·4 | 11·2 | 11·6 | 11·4 |
| | 3 | 8·8 | 8·4 | 8·6 | 8·6 |
| Urea | 1 | 5·7 | 5·6 | 5·4 | 5·8 |
| (mmol/l) | 2 | 15·5 | 15·6 | 15·0 | 15·3 |
| | 3 | 10·7 | 10·0 | 10·5 | 10·6 |
| Creatinine | 1 | 90·9 | 89·0 | 90·0 | 91·0 |
| (μmol/l) | 2 | 563·6 | 564·0 | 562·0 | 563·0 |
| | 3 | 327·3 | 325·8 | 326·0 | 327·4 |
| AST (U/l) | 1 | 12·0 | 11·0 | 12·0 | 12·0 |
| | 2 | 81·0 | 83·0 | 80·6 | 80·8 |
| | 3 | 46·8 | 47·0 | 45·9 | 46·3 |
| ALT (U/l) | 1 | 11·0 | 11·0 | 11·0 | 11·0 |
| | 2 | 80·0 | 80·0 | 79·4 | 80·0 |
| | 3 | 46·2 | 45·4 | 46·0 | 47·0 |
| Alkaline | 1 | 35·0 | 34·6 | 35·4 | 35·0 |
| phosphatase | 2 | 92·0 | 91·6 | 91·4 | 92·2 |
| (μ/l) | 3 | 65·0 | 66·0 | 63·0 | 64·7 |

1, physiological level; 2, pathological level; 3, mixture of 500 μl of 1 and 500 μl of 2 [3].

*Analytical*

Evaluation of each analyser lasted for between six and eight weeks. Analytical methods used are shown in table 1(*b*). Each analyser had built-in thermostat control device for assays that required incubation at 37°C. Accuracy of results were studied using the department's internal quality control sera (Biotol, rue de Foin, Paris, France). Imprecision was assessed with the same internal quality control sera in duplicate at the beginning and end of each batch, and at irregular intervals between test patients' within-batch samples. A third QC serum was made by mixing equal aliquots of the internal QC sera [3]; this was assayed in a similar way. The theoretical value of this third QC serum was calculated from the sum of the physiological and pathological sera divided by two (see table 1[*a*]) (Biotol, rue de Foin, Paris, France).

The effect of high concentrations of bilirubin and lipids were evaluated using highly icteric and turbid samples chosen randomly from patients' specimens. The throughput time of each analyser was studied by taking the average time to assay, in triplicate, 12 identical parameters.

Two technicians with different levels of experience in clinical laboratory work (about 3 months and over 2 years, respectively) were shown how to operate each analyser. They were then each given 50 randomly chosen samples to assay for glucose, urea, creatinine, AST, ALT and alkaline phosphatase on each analyser on 2 consecutive days.

*Table 1(b). Analytical methods used during this study.*

| | Hitachi 705 | EPOS 5060 | ERIS 6170 | Analyst |
|---|---|---|---|---|
| Glucose | Hexokinase/ G-6P-DH[6] | Gluc/ DH | Gluc/ DH | Hexokinase/ G-6P-DH[6] |
| Urea | Urease/ GLDH | —DO— | —DO— | —DO—[7] |
| Creatinine | Jaffé kinetic | —DO— | —DO— | Creatinine amidohydrolase[8] |
| AST | Aspartate | —— | NAD+[9] | AST/MDH[10] |
| ALT | Alanine | —— | NAD+[9] | pH 7·8 |
| Alkaline phosphatase | pNPP[11] | pNPP[11] | pNPP[11] | pNPP[12] |

*Table 2. Comparison of imprecision using Biotrol quality control serum (N = 50).*

| Analyte | Hitachi | EPOS | ERIS | Analyst |
|---|---|---|---|---|
| Glucose (mmol/l) | | | | |
| Mean | 5·3 | 5·2 | 5·2 | 5·3 |
| SD | 0·53 | 0·52 | 0·52 | 0·53 |
| % CV | 2·65 | 2·65 | 2·65 | 2·63 |
| Urea (mmol/l) | | | | |
| Mean | 5·4 | 5·6 | 5·6 | 5·8 |
| SD | 0·53 | 0·54 | 0·54 | 0·55 |
| % CV | 2·65 | 2·70 | 2·70 | 2·72 |
| Creatinine (μmol/l) | | | | |
| Mean | 90 | 91 | 90 | 91 |
| SD | 2·18 | 2·19 | 2·18 | 2·19 |
| % CV | 10·90 | 10·95 | 10·90 | 10·95 |
| AST (U/l) | | | | |
| Mean | 11·0 | 12·0 | 13·0 | 12·0 |
| SD | 0·75 | 0·79 | 0·83 | 0·79 |
| % CV | 3·80 | 3·95 | 4·15 | 3·95 |
| ALT (U/l) | | | | |
| Mean | 11·0 | 11·0 | 12·0 | 11·0 |
| SD | 0·76 | 0·76 | 0·79 | 0·76 |
| % CV | 3·80 | 3·80 | 3·95 | 3·80 |
| Alkaline phosphatase (U/l) | | | | |
| Mean | 33·4 | 33·0 | 34·0 | 33·0 |
| SD | 1·33 | 1·32 | 1·33 | 1·32 |

*Table 3. Coefficient of within-batch variation.*

| Analyte | | Biotrol Int. QC (Hitachi) | Merck QC (ERIS) | Du Pont QC (Analyst) |
|---|---|---|---|---|
| Glucose | N | 0·7 | 0·9 | 0·8 |
| | P | 0·8 | 0·9 | 0·8 |
| Urea | N | 1·9 | 1·8 | 1·7 |
| | P | 0·7 | 0·9 | 0·8 |
| Creatinine | N | 1·1 | 1·2 | 1·2 |
| | P | 1·2 | 1·2 | 1·2 |
| AST | N | 0·7 | 0·9 | 0·8 |
| | P | 2·1 | 2·5 | 2·2 |
| ALT | N | 1·5 | 1·8 | 1·4 |
| | P | 3·6 | 3·2 | 3·3 |
| ALP | N | 2·5 | 2·4 | 2·6 |
| | P | 2·6 | 2·3 | 2·6 |

N, physiological; P, pathological.

*Correlation studies*

Fifty randomly selected test patient samples from the daily pool were assayed simultaneously on each analyser, and on the Hitachi 705, for glucose, urea, creatinine, AST, ALT and alkaline phosphatase in triplicate for 5 consecutive weeks. The cumulative average value for each parameter from each analyser was calculated. Aliquots of six samples from the ERIS 6170 study kept at about −20°C for around three weeks were thawed and assayed for creatinine only on the Analyst.

Scatter diagrams of correlations using the calculated cumulative average values, were plotted between the Hitachi 705 and ERIS 6170, Hitachi 705 and Analyst and ERIS 6170 and Analyst, respectively, for the parameters indicated. The correlation coefficients (and regression lines) were calculated according to Swinscow [4].

### Results

Table 1(*a*) shows the variation in the mean accuracy values quoted for the quality control (Biotrol) sera for the parameters indicated between the three autoanalysers tested. On the basis of these results, accuracy can be classified as good. Imprecision was also, on average, satisfactory, see table 2. The dynamic throughput times are given in table 6. Although the times observed during this study are slightly longer than the manufacturers' claims, they are quite satisfactory.

Correlation scatter plots between the Hitachi 705 and ERIS 6170 and Hitachi 705 and Analyst are shown in figure 1; and those between the ERIS 6170 and Analyst in figure 2 for the parameters indicated. The corresponding calculated correlation coefficients are shown in tables 4 and 5. Good linear positive correlations were observed for the parameters indicated. The correlation observed for creatinine between the ERIS 6170 and the Analyst is interesting since the study was not carried out simultaneously on the same day, as were those between the Hitachi 705 and ERIS 6170, and Hitachi 705 and Analyst.

*Table 4. Correlation results from the Hitachi 705 compared with those of the Analyst and ERIS 6170 (N = 50).*

| | Slope | | Intercept ($y$)† | | Correlation coeff. ($r$) | | Standard error ($t$) | | P |
|---|---|---|---|---|---|---|---|---|---|
| | A | E | A | E | A | E | A | E | A E |
| Glucose | 1·0 | 0·9 | 19(1·9) | 27(2·1) | 0·99 | 0·89 | 0·004 | 0·004 | 0·001 |
| Creatinine | 0·9 | 1·0 | 0·19(0·1) | 0·2(0·14) | 0·99 | 0·98 | 0·004 | 0·004 | 0·001 |
| Urea | 1·5 | 1·3 | 15(3·4) | 19(2·7) | 0·98 | 0·98 | 0·004 | 0·004 | 0·001 |
| AST | 1·0 | 1·0 | 2 × (1·8) | 3 × (1·9) | 0·99 | 0·98 | 0·004 | 0·004 | 0·001 |
| ALP | 0·9 | 0·9 | 10 × (6) | 9 × (7) | 0·99 | 0·99 | 0·004 | 0·004 | 0·001 |
| ALT | 1·3 | 1·3 | 3(3) | 4(3) | 1·05 | 1·03 | 0·004 | 0·004 | 0·001 |

† Units as in tables 1(*a*) and 2; P, Probability distribution of $t$ [7]; A, Analyst; and E, ERIS 6170.

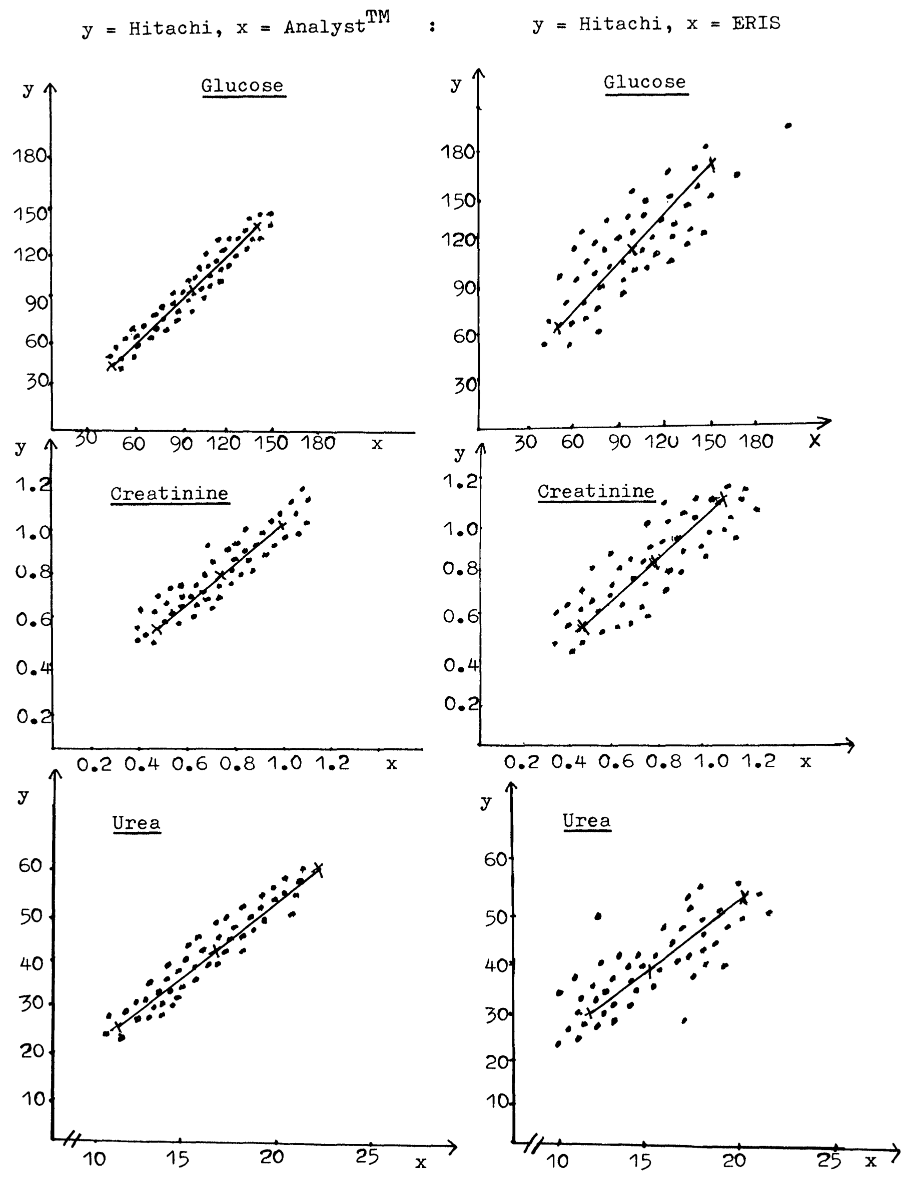

*Figure 1(a). Scatter diagram to show the correlation between the Hitachi 705 and the Analyst, and the Hitachi 705 and the ERIS 6170. N = 50.*

*Table 5. Correlation results from the ERIS compared with those from the Analyst for creatinine.*

| Slope | Intercept ($y$)† | Correlation coefficient ($r$) | Standard error ($t$) | P |
|---|---|---|---|---|
| 1·0 | 0·1 | 1·0 | 0·003 | 0·01 |

† Units as in tables 1 and 2; P, distribution of $t$ [7].

*Table 6. Dynamic throughput times: number of analyses per hour.*

| | Manufacturer's claim | This study results |
|---|---|---|
| EPOS | 300 | 270 |
| ERIS | 400 | 360 |
| Analyst | 55 | 50 |

### Discussion

All the analysers evaluated in this study require separated serum for analyses. Both the EPOS 5060 and ERIS 6170 can be modified to carry out other non-routine tests. The Analyst, on the other hand, can only assay for those tests indicated in any one chem disc rotor provided by the manufacturer. All three analysers are much more economical in terms of the sample and reagent volumes required per test, than are many other analysers such as the ACA III (a second autoanalyser now used for stat and drug analyses in the department), which requires about 150 μl of serum per test.

A preprogrammable sample dilution facility is available with the POS 5060. This facility was very useful in making 1 : 10 and or 1 : 20 dilutions for samples which indicated over the linear assay limits, and for imprecision and accuracy studies. A similar facility was not available for either of the other two analysers.

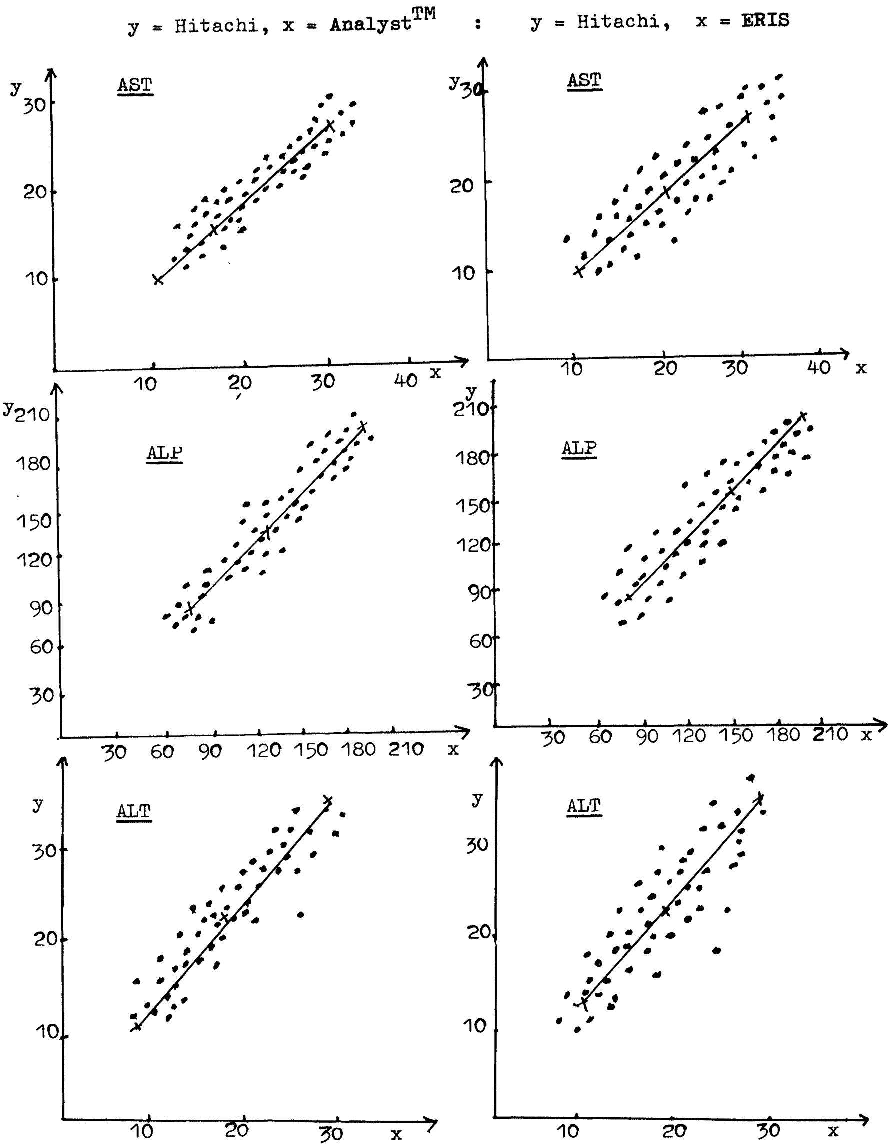

*Figure 1(b). Scatter diagram to show the correlation between the Hitachi 705 and the Analyst; and the Hitachi 705 and the ERIS 6170. N = 50.*

Imprecision, on the whole, was best in the Analyst, even with highly icteric and lipaemic samples. In general, imprecision was observed to be very consistent in all three analysers. The within-batch imprecision values, shown in table 2, are satisfactory for the three analysers.

The overall performance of the three analysers was quite satisfactory. Practically, the Analyst, a bench-top analyser would be preferred in Saudi's 'polyclinics' and in small paediatric laboratories. The Analyst is compact, portable and much easier to operate, than either of the other two analysers. The suitability of the Analyst for the UK's National Health Service Health Centres has also been discussed in *Mims Magazine* [5] (the Health Centres are similar to the 'polyclinics').

The EPOS 5060 would be suitable for hospital laboratories which serve daily disease-related clinics, for example diabetic clinics. The ERIS 6170, on the other hand, is a reliable analyser that could prove invaluable in any large routine clinical chemical laboratory.

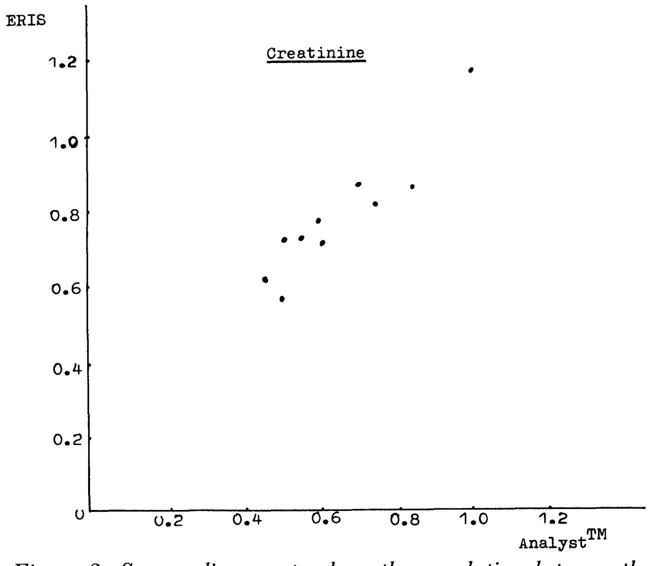

*Figure 2. Scatter diagram to show the correlation between the ERIS 6170 and the Analyst.*

### Acknowledgements

I wish to thank Mr M. Malgapo and Miss Kim for their willingness to help with the assessment of technician training time. I also wish to thank Mr R. Alam (Du Pont) and Mr K. D. Oberschmidt (Eppendorf Geratebau) for their technical assistance.

### References

1. Alpert, N. L., *Clinical Instrument Systems*, **6** (1985), 1.
2. Callagham, S. J., Singer, R., White, J. M. and Fraser, C. G., *Journal of Automatic Chemistry*, **7** (1985), 90.
3. Barnett, R. N. and Pinto, C. L., *American Journal of Clinical Pathology*, **48** (1967), 243.
4. Swinscow, T. D. V., *Statistics at Square One* (British Medical Association, London, 1978).
5. *Mims Magazine* (15 October, 1987), **1**, 20.
6. Slein, M. W., *Methods of Enzymatic Analysis*, Ed. Bergmeyer, H. U. (Academic Press, New York, 1965), 117.
7. Talke, H. and Schubert, G. E., *Klin Wochschur*, **43** (1965), 174.
8. Fossati, P., Prencipe, L. and Berti, G., *Clinical Chemistry*, **29** (1983), 1494.
9. Reitman, S. and Frankel, S., *American Journal of Clinical Pathology*, **28** (1957), 56.
10. Saris, N. E., *Clinical Chemistry*, **24** (1978), 720.
11. Bessey, O. A., Lowry, O. H. and Brock, M. J., *Journal of Biological Chemistry*, **164** (1947), 321.
12. Bowers, G. N. and McComb, R. B., *Clinical Chemistry*, **12** (1966), 70.

